# An overview on viral interference during SARS-CoV-2 pandemic

**DOI:** 10.3389/fped.2023.1308105

**Published:** 2023-12-21

**Authors:** Luigi Matera, Sara Manti, Laura Petrarca, Alessandra Pierangeli, Maria Giulia Conti, Enrica Mancino, Salvatore Leonardi, Fabio Midulla, Raffaella Nenna

**Affiliations:** ^1^ Department of Maternal Infantile and Urological Sciences, Sapienza University of Rome, Rome, Italy; ^2^ Department of Human and Pediatric Pathology, Pediatric Unit, G. Martino Hospital, University of Messina, Messina, Italy; ^3^ Laboratory of Virology, Department of Molecular Medicine, Affiliated to Istituto Pasteur Italia, Sapienza University of Rome, Rome, Italy; ^4^ Pediatric Respiratory Unit, Department of Clinical and Experimental Medicine, University of Catania, Catania, Italy

**Keywords:** viral interference, respiratory viruses, respiratory syncytial virus, RSV, SARS-CoV-2, COVID-19, influenza

## Abstract

Respiratory viruses represent the most frequent cause of mortality, morbidity and high healthcare costs for emergency visits and hospitalization in the pediatric age. Respiratory viruses can circulate simultaneously and can potentially infect the same host, determining different types of interactions, the so-called viral interference. The role of viral interference has assumed great importance since December 2019, when the severe acute respiratory syndrome coronavirus 2 (SARS-CoV-2) came on the scene. The aim of this narrative review is to present our perspective regarding research in respiratory virus interference and discuss recent advances on the topic because, following SARS-CoV-2 restrictions mitigation, we are experimenting the co-circulation of respiratory viruses along with SARS-CoV-2. This scenario is raising many concerns about possible virus-virus interactions, both positive and negative, and the clinical, diagnostic and therapeutic management of these coinfections. Moreover, we cannot rule out that also climatic conditions and social behaviours are involved. Thus, this situation can lead to different population epidemic dynamics, including changes in the age of the targeted population, disease course and severity, highlighting the need for prospective epidemiologic studies and mathematical modelling able to predict the timing and magnitude of epidemics caused by SARS-CoV-2/seasonal respiratory virus interactions in order to adjust better public health interventions.

## Introduction

Respiratory viruses represent the most frequent cause of significant morbidity and high healthcare costs for emergency visits and hospitalization worldwide, in particular in the pediatric age (1). In particular, respiratory syncytial virus (RSV) it is the most common cause of bronchiolitis, followed by rhinovirus (RV), and several others respiratory viruses, influenza A and B virus (IV), metapneumovirus (MPV), parainfluenza virus (PIV) 1–3, the endemic coronaviruses (CoV) OC43, 229E, NL-63 and HUK1, adenovirus and bocavirus (BoV) (2).

Several respiratory viruses can circulate simultaneously and can potentially infect the same host, determining different types of interactions. At the same time, environmental factors contribute to the ecology of the respiratory tract microbiome and determine viral infectivity; among others are the nutrient availability, oxygen tension, pH, temperature and human immune system interactions (3). Viral interference was originally defined as a state of temporary immunity due to an infection with a virus that limits the replication of a second infecting virus (4). Negative (i.e., antagonistic) virus–virus interaction can be homologous or heterologous, depending on whether interacting viruses belong to the same family or not. Homologous virus–virus interaction leads to cross-reactive immunity between the two viruses that prevents the infection with the second virus, for example different influenza subtypes (5). Heterologous viral interference is determined by the onset of a non-specific innate immune response by the first virus that reduces or prevents the infection by the second virus, such as influenza A virus and RSV (6).

Viral interference has been described long ago for different bacterial and animal viruses (4). Interestingly, in 1960 (7), researchers found that attenuated enterovirus infections in children prevented not only enteric pathogens, but decreased also detection of several respiratory viruses, such as IV, PIV, RSV, RV and adenovirus. Thus, they speculated that this effect could be mediated through the interferon-inducing effect of non-pathogenic enteroviruses. In fact, the activation of the innate immunity makes the respiratory mucosal cells resistant to a second virus replication, through the interferon response (8, 9).

**Figure 1:**
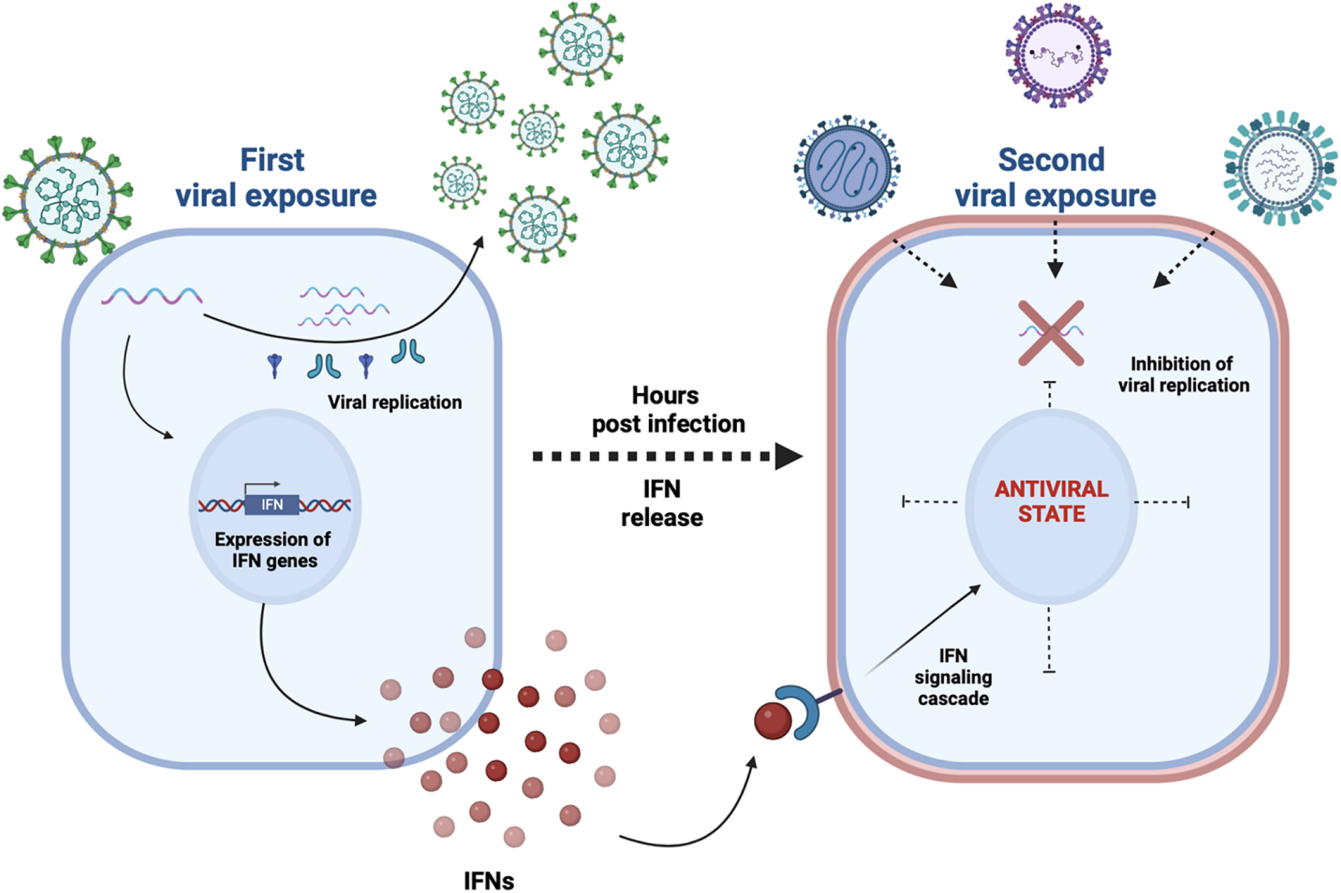
A first viral exposure results in a productive viral infection and spread within the human respiratory epithelium; a type I and III interferons (IFNs) response triggered by specific viruses (e.g. rhinoviruses) makes most cells transiently nonpermissive to subsequent infections (e.g. SARS-CoV-2, influenza A virus) as detailed in references (9–11).

Since then, different studies (9–11) demonstrated that a respiratory viral infection could prevent the infection of other respiratory pathogens, in particular due to the activation of the innate immunity (Figure 1).

Several epidemiological studies have provided data supporting the importance of viral interference in the epidemiology of respiratory viruses. For example, the circulation of influenza virus H1N12009 during the first pandemic winter has been delayed by RV in September-October 2009, in several countries (11, 12). Similarly, during the 2009 influenza pandemic, a delay in the RSV season onset was reported (13). Moreover, RV circulation in autumn can also influence RSV epidemic seasons (14, 15).

Since December 2019, the severe acute respiratory syndrome coronavirus 2 (SARS-CoV-2) came on the scene. It was declared a pandemic on 11 March 2020 (16). SARS-CoV-2 transmission primarily occurs through aerosolized droplets (17, 18), even if a fecal-oral transmission may also occur (19, 20). Thus, children can be infected through close contact with a sick family member (21) or schoolmate (22, 23).

Two Italian multicenter studies showed a sharp decrease in hospital admissions for respiratory viruses since the beginning of the SARS-CoV-2 pandemic: an 88.5% drop between March and May 2020, when the restrictive measures were more stringent, and a 64% drop between September and November 2020 (24, 25). Furthermore, we showed that respiratory viruses' circulation was drastically reduced from March 2020 to February 2021. This reduction involved RSV, influenza, viruses A and B, coronaviruses OC43, 229E, NL-63 and HUK1, adenoviruses, parainfluenza viruses 1–3, bocavirus and metapneumovirus, with exception of rhinovirus, that showed an incidence peak in September and October 2020 during the reopening of schools (26). Subsequently, we demonstrated an anticipated RSV circulation in September 2021 with a southward spread, from the north to the south of Italy. And RSV incidence was drastically reduced in January 2022 when the Omicron B.1.1.529 variant of SARS-CoV-2 started to circulate in pediatric patients (27). All these findings confirmed that SARS-CoV-2 may present viral interference with other common respiratory viruses.

Here, we present our perspective regarding research in respiratory virus interference and discuss recent advances on the topic. We particularly discuss the emerging evidence on the relationship between SARS-CoV-2 and Influenza and Respiratory Syncytial Virus. Finally, we speculate on the implications of this altered viral ecology on the clinical landscape in terms of impact on the healthcare system. In fact, several respiratory viruses can circulate during the same season, and they can concurrently or sequentially infect the respiratory tree, resulting in positive (additive or synergistic) or negative (antagonistic) virus‒virus interactions (28). While the positive virus‒virus interaction results in more aggressive pathogenesis and disease severity (e.g., SARS-CoV-2 and influenza A-H1N1) (28, 29), negative virus‒virus interaction can be homologous or heterologous. Occurring in viruses belonging to the same family, the homologous virus‒virus interaction makes that the cross-reactive immunity against the first virus prevents infection with the second virus (6, 30, 31). Whether the virus‒virus interaction involves two viruses belonging to different families, the aspecific innate immune response induced by the first virus can decrease or prevent the infection and replication of the second virus. Consequently, the infections that occur concurrently or sequentially may influence the epidemic behavior of each virus as well as the course and severity of disease in the host (29). Since several factors can interfere with the dynamic virus-virus interaction, the result is difficult to predict. The understanding of virus-virus interaction needs to clarify transmission models of respiratory viruses, virus shedding time, the capacity of the first virus to induce a rapid immune response, the ability of the second virus to be susceptible to immune mediators and the efficiency of the immune system of the host. Lastly, ecosystem conditions (e.g., weather time, temperature, humidity) and social behaviors different for age groups (e.g., school closing, work closing) can also affect the virus–virus interaction.

Hence, different virus–virus interactions will lead to unpredictable epidemic seasons and will result in changes in the clinical expression involving potentially a different targeted and wider population, ranging from milder-to-severe disease. Moreover, these interactions can determine the emergence of new viral variants. Thus, viral interference raises many questions, in particular on prospective epidemiologic studies and mathematical modelling able to predict the timing and magnitude of epidemics caused by SARS-CoV-2/seasonal respiratory virus interactions, in order to adjust better public health interventions, such as the use of prophylactic agents, changes in vaccine schedules and implementation of non-pharmacologic measures.

## Materials and methods

The literature research was performed searching PubMed through the keywords “SARS-CoV-2”, “SARS-Cov2”, “COVID-19”, “COVID19”, “respiratory virus”, “respiratory infection”, “acute communicable disease”, “lung disease”, “lung infection” and “viral interference”. The articles in English published from 2020 to 2022 were included. Articles relating to subjects younger than 18 years were included. Articles were excluded by title, abstract, or full text for irrelevance to the investigated issue. Specific types of articles such as clinical cases, editorials, letters or conference abstracts were excluded. Lastly, to identify further studies that met the inclusion criteria, the references of the selected articles were also reviewed.

## Results

During SARS-CoV-2 pandemic, a drastic reduction of respiratory virus circulation was registered worldwide (24–27, 32–36). Following the emergence of COVID-19 and in line with the implementation of public health measures, both individual (social distancing, masking, respiratory etiquette, screening, isolation and quarantine of the individuals, hand hygiene) and community (lockdown, school and work closures, contact tracing, reduction in public transport availability and use, public health education) interventions, a virtual absence from April 2020 to May 2021 of RSV activity has been described (26). After the first year of pandemic, a gradual increase in the circulation of respiratory viruses was noted, that found a susceptible pediatric population made up of both naïve children, who have never contracted a respiratory infection, and children that had been less frequently exposed to respiratory infections over the last 2 years. In a sort of mutual influence, viruses displayed different trends in circulation. For example, RV showed a peak in September and October 2020 during the reopening of schools, while the circulation of the other respiratory viruses was very reduced (25). Moreover, an early peak in RSV circulation was found in Italy followed by a drastic reduction in its circulation in January 2022, when the Omicron B.1.1.529 variant of SARS-CoV-2 began to spread in children (26). In parallel, a change in the age and severity of the primary infection of RSV were also reported (37, 38).

Intriguing evidence suggests a key role of viral interference in this extraordinary new picture of viruses' circulation. Viral interference may act in different ways (39), such as competitive survival, competitive binding to B-cells or inhibition of naïve B cells. Moreover, viral interference can play its role damaging/modifying the host's cellular receptors or enzymes required attachment, replication and survival of the second virus in the host cells (39, 40). There may occur a transient immunological response for the second virus infection after the first. Finally, memory B cells may increase antibodies' production against second viral infection (39, 40). The different responses after the first infection may protect the patient against the second virus or may increase the risk for severe disease, reinforcing the second infection. Different viruses show different way of interference. For example, influenza A(H1N1)pdm09 virus represent the most important inhibitor of subsequent viral infections (41). On the same way, rhinovirus infection was demonstrated to block SARS-CoV-2 replication in the respiratory epithelium (31).

### SARS-CoV-2 and influenza viruses

First observations showed that throughout the SARS-CoV-2 pandemic there was a 99% reduction in influenza virus isolation globally (42). The CDC influenza surveillance system reported a lower influenza infection incidence in 2020 (43). Moreover, several studies demonstrated that SARS-CoV-2 and influenza coinfections were rare (44). The most common co-infections with SARS-CoV-2 were RV, RSV and endemic coronaviruses, while influenza and adenoviruses represented the less frequent co-infections (45). The lower incidence of influenza may be explained by social distancing, ban of mass gathering and face masks but also by viral interference.

The first evidence of a possible viral interference between SARS-CoV-2 and influenza viruses comes from animal models: sequential infection of hamsters with H1N1 and SARS-CoV-2 determined a lower pulmonary SARS-CoV-2 load, suggesting a reduced lung replication (28). In contrast, previous infection with SARS-CoV-2 did not affect H1N1 lung load. Moreover, researchers found that in ferrets infected with influenza virus, non-specific immune response needed about 1–2 days to be elicited (46). During this period a co-infection with a second virus was likely to occur (46). Thereafter, the host innate immune response increased in 2–3 days and persists for 5–6 days, the period when viral interference occurs (46).

In human nasal epithelium cells, when the second infection occurred with a 48-hour delay, SARS-CoV-2s infection was slightly reduced by H1N1 first infection, while H1N1 infection 48 h after SARS-CoV-2 infection appeared to have no significant impact on the intracellular genome levels of influenza (47). In a context of successive infections with a much longer delay (7 days), was demonstrated that superinfection with SARS-CoV-2 had no significant impact on the intracellular influenza virus genome levels (47). Interestingly, comparing the immune system products during superinfections and those during the corresponding single infections, different profiles for influenza virus and SARS-CoV-2 were showed. In fact, during influenza infection with a subsequent SARS-CoV-2 superinfection, a significant upregulation of several genes was noted with exception of IL1A, IL1B, IL1R2, FAM89A, PTGS2, IL7R, MIP2α, IL18, and IL6 (47). In contrast, several genes involved in the interferon-stimulated innate immune response (OAS1, OAS2, SOCS1, DDX58, 16 CXCL10) were upregulated following influenza superinfection during SARS-CoV-2 infection (47). These observations suggest that the sequential order of infection is crucial for viral interference, with a higher antagonist of SARS-CoV-2 on influenza than vice versa (47).

Regarding Flu A and SARS-CoV-2 interference, Cheemarla et al. modeled this coinfection in human airway epithelial cultures. Flu A induced a more robust interferon response than SARS-CoV-2 suppressing SARS-CoV-2 replication in both sequential and simultaneous infections. On the other hand, SARS-CoV-2 did not induce this robust interferon response and did not suppress Flu A replication. Oseltamivir, a therapy targeting influenza virus, reduced Flu A replication during coinfection but on the same way reduced the host interferon response and restored SARS-CoV-2 replication (48).

Interestingly, a beneficial effect of flu vaccination on SARS-CoV-2 related mortality in the elderly (>65 years) was suggested (49), but no study in the pediatric population is available to our knowledge. There are conflicting studies concerning this aspect. Several studies demonstrated that influenza vaccinated patients were at higher risk for non-influenza viral infections than individuals directly infected by influenza virus, because they did not exhibit the non-specific immunity response associated with the natural infection (50–54). Concerning SARS-CoV-2 and flu vaccination interactions, there are contrasting results. In fact, while some studies found that flu vaccination correlated negatively with SARS-CoV-2 outcomes (55, 56), other studies found that flu vaccination were significantly associated with SARS-CoV-2 infection rates (57, 58).

Finally, in airway epithelial cells infected with rhinovirus, the influenza replication was reduced compared to control group and this suppression was not limited to Flu A, but involved also Flu B (59). Going deeper in the analysis, the authors showed that, in airway epithelial cells infected with rhinovirus, chemokines and cytokines (CXCL10, CCL5, CXCL9), interferons (IFNL1, IFNL2, IFNL3) and RNA processing factor (APOBEC3A) were upregulated. On the other hand, the genes BBS9, DYNC2H1, UNC119B, related to cilium assembly, resulted downregulated (59).

### SARS-CoV-2 and respiratory syncytial virus

RSV is an enveloped RNA virus that belongs to the family Pneumoviridae and the genus Orthopneumovirus (60). It is a significant cause of morbidity and hospital admission due to respiratory tract infections among infants and young children. Over 33 million episodes of RSV infections are reported globally in children below 5 years of age (61), and these episodes result in more than 3.2 million hospital admissions and about 120.000 hospital deaths worldwide (61).

Viral interference can alter the pattern of the infections; the simultaneous circulation of different viruses could create a competition with other respiratory viruses, change the epidemic dynamics, also delaying the usual epidemic peaks. Because RSV is horizontally transmitted by close contact, case counts decreased during the first COVID-19 pandemic period in almost all geographic regions and climate zones, thanks to the significant increase in public surveillance. Moreover, compared to the previous years, reports from the United States, Finland, United Kingdom, Belgium, and Italy showed a sudden and earlier end of the RSV season starting from March 2020, and almost no cases were notified in the following months (62–65). Even following the easing of restrictions, no RSV cases had been reported in Western Australia (66). In line with this, unusual epidemic peaks were recorded during atypical seasons of the year (37). Whether RSV seasonal peaks generally occurred during the autumn and winter seasons, peaks in the spring and summer were noted in Australia, South Africa, Brazil, and United States (38, 66–68). In Japan, RSV epidemics occurred earlier, and seasonal outbreaks were also observed during summer (69). This temporal shift was also reported in Spain, with the RSV epidemic onset in spring and no cases in the autumn and winter of 2020–2021 (70). Similarly, a prospective, observational, multicenter study involving four Italian Pediatric University Hospitals representative of the North, Center, and South of Italy showed that the RSV epidemic season occurred earlier than in the pre-pandemic year. Moreover, the RSV circulation followed a behaviour opposite to the SARS-CoV-2. While RSV outbreaks typically occurred in early autumn, a low SARS-CoV-2 activity was registered; conversely, when RSV reduced its incidence, the novel Omicron (B.1.1.529) variant of SARS-CoV-2 started to circulate among the pediatric population (27, 71). Supporting the evidence of viral interference, authors found that the rates of RSV/SARS-CoV-2 co-infections were lower than in previous seasons, which typically range from 8% to 12% (73, 74).

Looking at the RSV infection, 70% out of the global pediatric population is considered to have been infected at least once before the age of 2 years, and RSV reinfections may occur in otherwise healthy children but with no severe consequences or in adults aged 65 and older (74).

Comparing three consecutive years, the pre-pandemic clinical characteristics of RSV infection were different from the ones occurring during and post-pandemic COVID-19. During the COVID-19 pandemic, a sharp decrease in RSV infection was reported, in line with the implementation of lockdown and physical distancing measures; successively, an increase in the frequency of RSV infection with a milder disease course was reported (75–77). Due to the absence of RSV circulation, an increased number of children older than 2 years of age susceptible to RSV infection was notified (78, 79). Moreover, when hospitalized, the clinical symptoms of RSV patients appeared less severe, and respiratory support and oxygenation were less frequently needed (75). Accordingly, in the Italian multicenter study (27), the clinical respiratory score (CRS) assigned at the admission time of 587 children suffering from respiratory tract infections was predominantly mild (54%). In contrast, 39.7% of patients showed moderate CRS, and only 6.3% of children experienced severe CRS (27).

However, RSV infection's disease course and severity did not show a similar trend among different countries. Although the weekly number of hospital admissions registered during the epidemic was lower than in previous years, the post-pandemic clinical characteristics of the Spanish children were similar to previous epidemic seasons (70). In Australia, although reports revealed a decrease in epidemic RSV, the infection was associated with increased morbidity among patients aged 2–4 years, including outpatients (75). In their retrospective study conducted from January 2019 to December 2021 in China, authors reported that RSV infection remained a significant cause of admission to the emergency department in children under 1 year old, with a gradual increase in hospital rate also in children older than 1 year (80).

Several hypotheses have been formulated to explain these phenomena and the differences in the course and severity of RSV infection. Since RSV shows the same route of infection of SARS-CoV-2, the adopted restrictive measures to counteract the pandemic have impacted RSV transmission, justifying the virus's disappearance during the COVID-19 pandemic (80). Social distancing, mandatory facemasks, and hand hygiene reduced RSV transmission through droplets and contact through dirty hands and fomites (81, 82). The role of school closure and international borders limitation in reducing RSV transmission appeared controversial. The school reopening and borders reopening were not associated with an increase in RSV infections, probably, because even when children came back to school and the opening of international borders restarted, restrictive measures, including social distancing and hand hygiene, were maintained (83–86). Accordingly, children reporting mild respiratory symptoms were not admitted to school, reducing the circulation of other respiratory viruses. In their systematic review, authors reported that the implemented public health and social measures to counteract the spread of SARS-CoV-2 resulted in an overall decrease of 23%–94% in the incidence of respiratory viral diseases and a decrease of 0%–98% in the detection of the respiratory viruses (86). The interaction between environmental conditions, including weather and air pollution, and incidence of RSV has been also frequently described (87). Climate factors influence the spread of infection. RSV transmission seems to be positively associated with lower temperatures and higher relative humidity rates; moreover, adverse climate conditions make people stay at home and/or crowded places leading to an increase in RSV infection rate (88). Therefore, the differences detected in RSV incidence during and post-pandemic COVID-19 among countries should be evaluated also in light of contributions and correlations between RSV and climate parameters. The climate changes can be influenced by numerous environmental factors, such as air pollution. A ten-year prospective and observational study on infants aged less than 12 months showed a significant correlation between RSV-mediated bronchiolitis incidence and air pollution in an urban Italian area (87). COVID-19 lockdown changed the air quality with a significant decrease in air pollutants (benzene and nitrogen oxides) (89); thus, a role of reduced air pollution in the epidemiology of RSV should be also considered.

Adults and children with immunodeficiency are the major reservoirs for RSV; the adoption of non-pharmacological measures, especially in these clusters of population, might have contributed to reducing transmission in the pediatric population (70). It is also possible that the COVID-19 emergence affected RSV genomic variability since a significant reduction in RSV genetic diversity and aggressivity has been described and associated with a milder course disease (89, 90). Accordingly, even when RSV was identified in children after the relaxation of public health measures, the expected increases in infections and hospitalizations, especially during epidemic season, were not recorded, probably still reflecting adherence to public health measures (91).

Conversely, in their observational, epidemiological study, authors described a severe clinical phenotype in children affected by RSV/COVID-19 coinfections. Interestingly, it appeared that clinical outcomes were worse in children with convalescent SARS-CoV-2 and acute or convalescent RSV infection, showing a tight correlation between the viral loads (72).

Lastly, introducing new and complete molecular panels in the clinical practice made it possible to identify the causative agents, allowing early isolation of the affected patients and preventing the onset of epidemiological outbreaks (82, 92). On the other hand, multiplex PCR should be reserved for patients with symptoms and histories of sick contacts since their routine use for all inpatients might lead to an inappropriate management of positive cases since the FilmArray cannot quantify microorganisms (93).

### Limitation of the study

This manuscript is not a systematic review. It is a narrative review to present our perspective regarding research in respiratory virus interference and recent advances on this topic. We included articles in English, published from 2020 to 2022 and related to pediatric age. Finally, we excluded clinical cases, editorials, letters or conference abstracts were excluded.

## Conclusions

Following the COVID-19 pandemic and the gradual worldwide release of sanitary restrictions, we face the co-circulation of respiratory viruses together with SARS-CoV-2. This situation raises many concerns about the mechanisms of virus-virus interaction, the consequences of these co-infections/superinfections, and the clinical, diagnostic and therapeutic management of this unprecedented situation. The result of these different viral interactions is difficult to predict because we have to consider the transmission of respiratory viruses, the virus shedding time, the host immune response. Also, climatic conditions (e.g., weather time, temperature, humidity) and social behaviours different for age groups (e.g., school closing, work closing) are involved. Thus, the simultaneous circulation of different viruses can lead to different population epidemic dynamics, including changes in the age of the targeted population, disease course and severity.

In conclusion, prospective epidemiologic studies and mathematical modelling able to predict the timing and magnitude of epidemics caused by SARS-CoV-2/seasonal respiratory virus interactions are more urgent than ever to adjust better public health interventions, such as the use of prophylactic agents, changes in vaccine schedules and implementation of non-pharmacologic measures.
